# The risk of hemorrhagic complications in hospital in-patients who fall while receiving antithrombotic therapy

**DOI:** 10.1186/1477-9560-3-1

**Published:** 2005-01-07

**Authors:** Andrew J Bond, Frank J Molnar, Marilyn Li, Marlene Mackey, Malcolm Man-Son-Hing

**Affiliations:** 1 Clinical Epidemiology Program, Ottawa Health Research Institute, Ottawa, Ontario, Canada; 2 Elisabeth Bruyere Research Institute, Sisters of Charity Ottawa Health Service, Ottawa, Ontario, Canada; 3 Division of Geriatric Medicine, University of Ottawa, Ottawa, Ontario, Canada; 4 Department of Nursing Professional Practice, The Ottawa Hospital, Ottawa, Ontario, Canada

## Abstract

**Background:**

The use of antithrombotic agents and falls are independently associated with an increased risk of hemorrhagic injury. However, few studies have delineated the risk of fall-related hemorrhagic complications in persons who are taking antithrombotic therapy. The objective of this study was to compare the rates of fall-related hemorrhagic injury in hospital in-patients who are taking and not taking antithrombotic therapy.

**Methods:**

A 4-year retrospective chart review of consecutive patients who fell during admission to a 500-bed tertiary-care teaching hospital was conducted. Major hemorrhagic injuries including subdural hematomas and major bleeding/cuts, patients' use of antithrombotic medication (warfarin, aspirin, clopidogrel and heparin) and their anticoagulation status at the time of their fall were recorded.

**Results:**

A total of 2635 falls in 1861 patients were reviewed. Approximately 10% of falls caused major hemorrhagic injury. One fall resulted in a subdural hematoma. Persons taking warfarin were less likely to suffer a fall-related major hemorrhagic injury compared with persons not taking antithrombotic therapy (warfarin, 6%; no therapy, 11%; p = 0.01). Logistic regression showed that fall-related major hemorrhagic injury was associated with female gender (odds ratio 1.6; 95% CI 1.3, 2.1), use of aspirin (odds ratio 1.4; 95% CI 1.1, 1.8) and use of clopidogrel (odds ratio 2.2; 95% CI 1.1, 4.8), but not with the use of warfarin or heparin, or the intensity of anticoagulation.

**Conclusions:**

In this study, compared with persons taking no antithrombotic therapy, those taking warfarin had lower rates of fall-related hemorrhagic injuries. The absolute rate of the development of fall-related intracranial hemorrhagic injury such as subdural hematomas was low, even in persons taking warfarin. These counter-intuitive results may be due to selection bias, and suggest that physicians are very conservative in selecting patients for warfarin therapy, choosing only those who are sufficiently healthy to be at much lower than average risk of suffering fall-related hemorrhagic injuries. This phenomenon may lead to physicians overestimating the potential for fall-related major hemorrhagic injury in persons taking antithrombotic therapy, with the possible denial of warfarin therapy to many of those who would benefit. This perception may contribute to the care gap between the number of patients who would theoretically derive overall benefit from warfarin therapy and those who are actually receiving it.

## Introduction

Antithrombotic agents such as warfarin, aspirin, clopidogrel and heparin have proven efficacy and are widely prescribed in the prevention and treatment of many cardiovascular and cerebrovascular diseases [1-3]. However, a significant disadvantage of the use of these therapies is an increased incidence of major hemorrhagic episodes [4]. Compared to younger persons, those over the age of 65 years are at higher risk of these antithrombotic-related complications [5]. Falling, which is also more common in older persons, can also lead to an increased risk of hemorrhagic injury [6]. Thus, at least two studies [7,8] have attempted to delineate the risk of fall-related hemorrhagic complications in older persons who are taking antithrombotic therapy. Stein *et al *[7] examined 400 consecutive falls in stroke patients admitted to a rehabilitation center. They found no excess risk of hemorrhagic complications in those who were taking warfarin compared with those who were not. More recently, a study [8] using decision analytic modeling also determined that the risk of fall-related hemorrhagic complications was low, even in those taking warfarin. It concluded that the risk of falling should not influence the choice of antithrombotic therapy in older persons with atrial fibrillation. However, many clinicians continue to perceive that older persons who are at increased risk of falling have an unacceptably high risk of antithrombotic-related major hemorrhage [9]. Thus, some continue to withhold antithrombotic therapy in patients who could potentially derive significant benefit from treatment [10]. The objective of this retrospective study was to determine whether there is a difference in hemorrhagic complications in hospital in-patients who fall and are taking antithrombotic therapy compared with those who are not.

## Methods

The protocol was approved by the Ottawa Hospital Research Ethics Committee and is in compliance with the Helsinki Declaration. Since implementation of a fall prevention program in 1991, the Ottawa Hospital – Civic Campus, a 500-bed tertiary care teaching hospital, has had a formal policy of documenting the pertinent details of all patient-related falls occurring within the hospital. Using its dedicated falls database, the records of consecutive falls occurring over a 4-year period (January 1, 1997 to December 31, 2000) were identified. The hospital policy requires completion of an incident report when patients fall. Information collected includes the time and circumstances of the fall, the type of injury (including head injury) that occurred and a categorization of its severity, and the need for subsequent follow-up. A member of the physician team must eventually review all fall incident reports, follow-up any significant injury from the fall and document this information in the hospital record and incident report.

Hemorrhage (i.e. cuts or bruising) was classified as major if the immediate attention of a physician was required, or a clinically apparent intracranial hemorrhage subsequently occurred. All other hemorrhage was classified as minor.

Since patient falls can be seen as a reflection of sub-optimal nursing care, the hospital has adopted a non-punitive approach to the completion of reports in order to focus on improving the process that led to the falls. The hospital recognizes that the value of the falls database is predicated on maximum completion of incident reports, leading to the assignment of nurse practitioners dedicated to ensuring the completion of the falls reports. Therefore, it is unlikely that falls leading to an injury would not be captured. During the study period, the hospital had no formal policy or guidelines governing the use of antithrombotic therapy in patients.

For all identified falls, the hospital records of the corresponding in-patient admission were retrieved. Data extraction from these charts included pertinent patient demographic information, and the use of antithrombotic therapy (warfarin, aspirin, clopidogrel and/or heparin) at the time of the fall. The indications for the use of the particular antithrombotic therapy were also recorded. Using computerized laboratory reports, the international normalized ratio (INR) and partial thromboplastin time (PTT) values that were closest to the time of the fall were also recorded. Patients' INR values at the time of the fall were determined as follows:

1. If an INR had been done within 12 hours pre- or post-fall, this was accepted as the INR value at the time of the fall. For patients with both 12 hour pre- and post-fall INRs, these values were averaged.

2. For patients not receiving warfarin, if an INR had not been done within 12 hours of the fall and the closest temporal INR was within the normal range (INR<1.2), this was accepted as the INR value occurring at the time of the fall. If the INR values were abnormal, then the pre- and post-INR values temporally closest to the time of the fall were averaged.

3. For persons receiving warfarin at the time of the fall, if no 12 hour pre- and post fall INRs were available, then the pre- and post-fall INR values done temporally closest to the fall were averaged.

4. For persons who did not have an INR while in hospital and were not receiving warfarin, their charts were reviewed for possible reasons to have an elevated INR (e.g. liver disease, coagulapathies). If these were not present, their INR were deemed to be normal (INR 1.0).

For patients taking heparin, an identical approach was used for determining the PTT values at the time of their fall.

The discharge summaries, nursing notes and medical notes of the hospital records were also reviewed for any immediate and subsequent complications due to the fall including head trauma, subdural hematoma, intracerebral hemorrhage, fractures, major and minor hemorrhage. Multiple falls by the same person were considered independent events. Data extractors were not blinded to the exposure status of patients or their outcomes.

Statistical analysis was performed using SPSS software version 10 (SPSS, Chicago, IL). Chi-square testing was performed to determine the relationship between individual demographic and clinical factors, and the occurrence of major hemorrhage. Step-wise forward logistic regression analysis was then performed with variables that had p-values < 0.20 on univariate analysis. Given that multiple statistical comparisons were performed, a p-value < 0.01 was considered statistically significant.

## Results

For the 4-year period, there was a total of 2664 recorded falls in 1861 patients. Despite numerous attempts, the corresponding hospital records could not be located for 29 (1.1%) of the falls. Thus, pertinent data were available and extracted regarding 2635 falls. A significant percentage (29.4%) of patients fell more than once during their admission. The average age of the patients was 71.5 years (SD 15.2; range 16–104), with most being male (55.2%).

### Antithrombotic use persons who fell

Table 1 (see additional file 1) shows the details of the antithrombotic therapy use of the patients who fell. Approximately 50% of the patients were taking some form of antithrombotic therapy (warfarin, aspirin, clopidogrel or heparin) at the time of their fall. Approximately 20% of patients had INR values that were higher than the normal range, with a similar number having PTT values outside the normal range. The most common reason for patients to be taking warfarin was stroke prevention in atrial fibrillation. The most common reasons for taking aspirin and heparin were prevention of myocardial infarction and deep vein thrombosis prophylaxis respectively. No patients with high INRs due to reasons other than warfarin use (e.g. liver failure) were found.

### Fall-related injuries

Table 2 (see additional file 2) shows the subsequent injuries due to the falls. Major hemorrhage (i.e. bruising and/or cuts requiring immediate attention from a physician) occurred with 10.7% of falls (n = 282). Only one fall resulted in the development of a subdural hematoma. This person was an 89 year old male who was taking warfarin 2 mg and aspirin 81 mg daily (both for stroke prevention in atrial fibrillation) at the same time. His INR and PTT at the time of the fall were 4.3 and 77 seconds respectively. It was documented that he suffered head trauma during the fall. The subdural hematoma was confirmed by CT scan of the head, and he subsequently died from his injuries.

There was also one fall possibly resulting in an intracerebral hemorrhage occurring in a 60 year old female. She also suffered head trauma from her fall, but was not taking any antithrombotic therapy, and her INR and PTT values were in the normal range at the time of the fall. She recovered from this injury, with no apparent sequelae.

The absolute rate of major hemorrhagic injury (i.e subdural hematoma, intracerebral hemorrhage and major bruising) was lower in persons taking warfarin, compared with those taking no antithrombotic therapy at all (warfarin, 6.2%; no therapy, 11.3%; p = 0.01). A comparison of major hemorrhagic injury between those with normal INR values (INR = <1.3) and those in the therapeutic range (INR 2–3) showed a strong trend towards fewer complications in the group with therapeutic INRs (normal INR, 10.1% (209/2066); INR 2–3, 6.9% (15/218); odds ratio 0.65, 95% CI; 0.38, 1.13; p = 0.15). A similar comparison between those with normal INRs and those with INRs between 3–5, showed no statistical difference in hemorrhagic complications between the two groups (normal INRs, 10.1% (209/2066); INR 3–5, 11.4% (9/79); odds ratio 1.14, 95% CI; 0.56, 2.32; p = 0.70).

Univariate analysis demonstrated a very strong relationship between gender and the occurrence of fall-related major hemorrhagic injury (i.e. subdural hematomas, intracerebral hemorrhages and major bruising), with females being much more likely to suffer one of these complications compared with males (13.3% (157/1181) versus 8.7% (126/1454); p < 0.001). Univariate analysis also showed that there were trends towards an increase in the occurrence of major hemorrhagic injury with increasing age (p = 0.04), increasing INR values at time of fall (p = 0.04), and the use of clopidogrel (p = 0.05) or aspirin (p = 0.20). There was no relationship between major hemorrhagic injury with PTT values at time of the fall (p = 0.27), the use of warfarin (p = 0.42) or the use of heparin (p = 0.62). Similar analyses using major bruising/cuts alone (and excluding subdural hematomas and intracerebral hemorrhages) yielded almost identical results. This was due to the occurrence of only one subdural hematoma and one intracerebral hemorrhage.

Logistic regression analysis showed that the factors important in the development of major hemorrhagic injury due to falls were female gender (odds ratio 1.6; 95% CI 1.3, 2.1), the use of aspirin (odds ratio 1.4; 95% CI 1.1, 1.8) and the use of clopidogrel (odds ratio 2.2; 95% CI 1.1, 4.8). Of note, increasing age was not an independent risk factor and there was no interaction between warfarin and aspirin use.

Repeating the analyses with exclusion of all recurrent falls in individuals (n = 1861) resulted in no significant differences in the results reported above.

Of note, fractures occurred in 1.4% of falls (n = 38), with 20 of these being hip fractures.

## Discussion

Falling is a common phenomenon in both hospitalized [11] and community-dwelling [12] older persons, with many falls leading to major hemorrhagic injury. Many physicians perceive that the concomitant use of antithrombotic therapy (especially warfarin) increases the chance of fall-related major hemorrhagic injury. This study documents the frequency of these injuries due to falling in hospitalized persons taking antithrombotic therapy and compares them to those who are not.

Numerous studies [13-15] have shown that the frequency of hemorrhagic injury is directly proportional to the intensity of anticoagulation. This study found a significant trend (p = 0.04) towards higher intensity anticoagulation status (as measured by INR values) leading to an increasing chance of the development of fall-related major hemorrhagic injury. However, when compared to persons with INRs in the normal range (INR <1.3), there was no trend suggesting that persons with INRs in the therapeutic range (INR 2–3) suffer more frequent major hemorrhagic injury. This suggests that persons with INRs in the therapeutic range are not at increased risk of suffering fall-related major hemorrhagic injury, with excess risk only in those with INRs above the therapeutic range.

The overall results of this study found that persons taking warfarin were less likely to suffer a fall-related hemorrhagic injury, compared to those taking no antithrombotic therapy. This counter-intuitive result may be due to selection bias. That is, physicians were very conservative in selecting patients for warfarin therapy, choosing only those who were robust enough to be at very low risk of suffering a fall-related hemorrhagic injury. Thus, physicians possibly overestimate the potential for major hemorrhagic injury in persons taking antithrombotic therapy, leading to the possible denial of warfarin therapy to many of those in whom warfarin would otherwise be indicated. This practice may contribute to the well-documented care gap between the number of patients who would theoretically derive overall benefit from warfarin therapy and those who are actually receiving it. [16,17] However, it must be remembered that other reasons for this care gap may exist. For example, since the risk of stroke from atrial fibrillation more of a long-term, rather than short-term clinical decision, the in-hospital physicians may have had a tendency to defer decision-making about anticoagulation to the primary care physicians of these patients.

In this study, only 1 SDH occurred as a result of the more than 2500 falls. Therefore, it was not possible to perform meaningful statistical analyses regarding the contributors to this complication. However, the results confirm that fall-related subdural hematomas are not common in older hospitalized persons, even if they are taking antithrombotic agents. That being said, the development of the single SDH found in this study was almost certainly related to the concomitant use of warfarin and aspirin, with an associated INR of greater than 4.0.

The reason(s) for this study finding that female patients have a greater risk of developing fall-related hemorrhagic injury is unclear. Age was not a factor as the mean age of female patients (71.6 years) was similar to male patients (71.5 years). Also, the percentages of female and male patients taking warfarin, heparin, clopidogrel or aspirin in this study were very similar. Other studies [18,19] have shown that females are more likely to suffer fall-induced injuries compared with males, though these studies included non-hemorrhagic injuries such as fractures. The relationship between gender and fall-related fractures is explainable by the higher prevalence of osteoporosis in the older female population.

The use of aspirin or clopidogrel is generally considered to be less likely to lead to major hemorrhagic injury when compared with the use of warfarin or heparin. Therefore, it was surprising to find that there was a weak, but statistically significant association between fall-related major hemorrhagic injuries and the use of aspirin or clopidogrel, but no such relationship with the use of warfarin or heparin. Again, this result may be due to selection bias, with physicians favoring the use of aspirin or clopidogrel over warfarin or heparin in persons who are less healthy and more prone to serious hemorrhagic injury if they fall.

There are a number of limitations to our study. Due to the retrospective design, it was not possible to apply standardized definitions and measures when determining the occurrence, severity and consequences of falls. Also, we examined the injuries related to hospital-based falls. Therefore, it is unclear whether our results are generalizable to other settings. However, the 10.7% rate of major fall-related hemorrhagic injury (SDH, ICH or major bruising/cuts) in this study is similar to previous hospital- and community-based studies [6,12] that found that approximately 5%–10% of falls result in serious injury. In our database, there were fewer falls causing minor hemorrhagic injury compared with major hemorrhagic injury. This suggests that there was likely underreporting of minor hemorrhagic injury due to falls. This may have occurred because, despite hospital policy, nurses were less inclined to complete incident reports for patients whom they believed had no potential sequelae to their falls. Many falls that resulted in little to no injury may not have been captured. Therefore, the results of our study are likely to overestimate, rather than underestimate, the rate of hemorrhagic injury in persons who fall. The methodology of this study would have been strengthened by reviewing all hospital admissions over the study period for the risk of fall-related injuries. The rate of falling in those receiving and not receiving antithrombotic therapy could then be determined. If those receiving warfarin were less likely to fall, then the conclusion that clinicians were reluctant to prescribe antithrombotic agents (especially warfarin) to patients they deemed at risk for falls would be strengthened. Unfortunately, resource considerations prevented us from taking this approach. It also would have been advantageous to have collected further information regarding potential confounders related to bleeding risk such as the presence of previous falls or fractures, and the amount of time patients were in hospital. However, some of this information was not or could not be reliably collected from the charts. This is because most primary care physicians often defer to the specialists to make these decisions. Finally, since there were persons who fell multiple times, one could argue with our assumption that each fall was an independent event. However, reanalysis of the data by including only the first recorded fall from each individual resulted in no significant changes to the results.

The study also has an important strength. Not all fall-related major hemorrhagic injury (especially subdural hematomas) is identifiable immediately after a fall. We were able to follow-up the sequelae of falling throughout the course of the patients' hospital admission. Therefore, it is unlikely that we failed to identify any serious consequences of falling in our study population.

## Conclusion

This study provides evidence that the absolute rate of the development of fall-related subdural hematomas is low, even in persons taking warfarin. Also, the lower than expected rate of fall-related hemorrhagic injury in persons taking warfarin suggests that physicians may overestimate the potential for fall-related major hemorrhagic injury in older persons taking antithrombotic therapy, leading to an overly conservative approach to assessing the risk of anticoagulant-related bleeding. This information may help close the care gap between the number of patients who would theoretically benefit from anticoagulant therapy and the number that actually receive it. Further study is necessary to delineate the characteristics of patients who are at high risk of developing fall-related hemorrhagic injury when taking antithrombotic therapy.

## Competing interests

The author(s) declare that they have no competing interests.

## Authors' contributions

MM and FM proposed the study. All authors participated in the design of the study and contributed to the drafting and revision of the manuscript. AB and ML conducted the chart reviews.

## Supplementary Material

Additional File 1 TABLE1Aug04revised.doc : this is Table 1 entitled, "Fall-related antithrombotic use" Click here for file

Additional File 2 TABLE2Aug04revised.doc : this is Table 2 entitled, "Consequences of falls" Click here for file
