# Supplementary material for: The risk of hemorrhagic complications in hospital in-patients who fall while receiving antithrombotic therapy
Source: Thromb J. 2005 Jan 7;3:1. doi: 10.1186/1477-9560-3-1 (PMC545051; doi:10.1186/1477-9560-3-1)
Supplement: Additional File 1 — TABLE1Aug04revised.doc : this is Table 1 entitled, "Fall-related antithrombotic use" [file 1477-9560-3-1-S1.doc]

**TABLE 1**. **Fall Related Antithrombotic Use^*^ (Total n = 2635)**

|  | **%** | **(n)** |
| --- | --- | --- |
|  |  |  |
| **Taking Warfarin** | 14.4 | (379) |
| Reason: (may be more than one) |  |  |
| AF | 42.7 | (161) |
| DVT | 11.6 | (44) |
| Perioperative DVT prophylaxis | 33.2 | (126) |
| Heart valves | 10.2 | (39) |
| Other | 8.9 | (34) |
|  |  |  |
| INR Status |  |  |
| ≤ 1.30 | 78.4 | (2066) |
| 1.31 – 1.99 | 9.9 | (260) |
| 2.00 – 3.00 | 8.3 | (218) |
| 3.01 – 5.0 | 3.0 | (79) |
| > 5.0 | 0.3 | (8) |
| unknown | 0.1 | (4) |
| Taking ASA | 27.9 | (734) |
| Daily Dose (mg): |  |  |
| 80-81 | 13.5 | (99) |
| 162 | 0.1 | (1) |
| 325 | 84.0 | (617) |
| 650 and greater | 2.4 | (17) |
| Reason: (may be more than one) |  |  |
| Atrial Fibrillation | 10.9 | (80) |
| DVT prophylaxis | 8.3 | (60) |
| Cardiac | 59.8 | (432) |
| Stroke Prevention | 24.0 | (177) |
| Other | 3.0 | (22) |
|  |  |  |
| **Taking Clopidogrel** | 1.7 | (46) |
|  |  |  |
| **Taking Heparin** | 20.1 | (530) |
| Type: |  |  |
| Low molecular weight | 5.7 | (30) |
| Unfractionated Fixed dose (5000 u) | 84.5 | (448) |
| Unfractionated Adjusted dose | 9.8 | (52) |
| Reason: |  |  |
| DVT | 3.0 | (16) |
| DVT prophylaxis | 77.0 | (408) |
| Heart | 3.7 | (98) |
| Stroke | 1.1 | (28) |
|  |  |  |
| PTT Status |  |  |
| < 35 sec | 68.4 | (1802) |
| 35-44 sec | 18.9 | (499) |
| 45-60 sec | 8.5 | (225) |
| >60 sec | 4.1 | (109) |
|  |  |  |
| **INR** – International Normalized Ratio |  |  |
| **PTT** – Partial Thromboplastin time |  |  |
| **DVT** – Deep Vein Thrombosis |  |  |

*Patients may have been taking more than one type of antithrombotic therapy
