# Supplementary material for: The risk of hemorrhagic complications in hospital in-patients who fall while receiving antithrombotic therapy
Source: Thromb J. 2005 Jan 7;3:1. doi: 10.1186/1477-9560-3-1 (PMC545051; doi:10.1186/1477-9560-3-1)
Supplement: Additional File 2 — TABLE2Aug04revised.doc : this is Table 2 entitled, "Consequences of falls" [file 1477-9560-3-1-S2.doc]

# TABLE 2. Consequences of Falls^*^ (n=2635)

|  | No Antithrombotics  (n=1216) | Warfarin (n=376) | **Aspirin** (n=734) | Heparin (n=530) | **Clopidogrel** (n=46) | **All**(n=2635) |
| --- | --- | --- | --- | --- | --- | --- |
| % hit head (n) | 18.8 (228) | 17.6 (67) | 17.8 (131) | 15.1 (80) | 30.4 (14) | 17.5(462) |
| % Subdural Hematoma (n) | 0.0 (0) | 0.03 (1) | 0.03 (1) | 0.0 (0) | 0.0 (0) | 0.0(1) |
| % Intracerebral Hemorrhage (n) | 0.0 (0) | 0.0 (0) | 0.0 (0) | 0.0 (0) | 0.0 (0) | 0.0(1) |
| % Major Hemorrhagic Injury (n) | 10.3 (125) | 7.4 (28) | 11.9 (87) | 7.7 (41) | 19.6 (9) | 10.7(282) |
| % Minor Hemorrhagic Injury (n) | 3.1 (38) | 2.7 (10) | 3.3 (24) | 2.8 (15) | 6.5 (3) | 3.5(91) |
| % Fracture (n) | 0.1 (13) | 2.4 (9) | 1.0 (7) | 2.1 (11) | 0.0 (0) | 1.4 (38) |
| Type of fracture: |  |  |  |  |  |  |
| hip (n) | 8 | 4 | 3 | 6 | 0 | 20 |
| vertebral (n) | 1 | 0 | 1 | 0 | 0 | 2 |
| other (n) | 5 | 5 | 3 | 5 | 0 | 17 |

*Patients may have been taking more than one type of antithrombotic therapy
